# Glutamate triggers the expression of functional ionotropic and metabotropic glutamate receptors in mast cells

**DOI:** 10.1038/s41423-020-0421-z

**Published:** 2020-04-20

**Authors:** Md Abdul Alim, Mirjana Grujic, Paul W. Ackerman, Per Kristiansson, Pernilla Eliasson, Magnus Peterson, Gunnar Pejler

**Affiliations:** 1 Department of Public Health and Caring Sciences, General Medicine and Preventive Medicine, Uppsala University, Uppsala, Sweden; 2 Department of Medical Biochemistry and Microbiology, Uppsala University, Uppsala, Sweden; 3 Department of Molecular Medicine and Surgery, Karolinska Institute, Stockholm, Sweden; 4 Department of Orthopedics and Sports Medicine, Linköping University, Linköping, Sweden; 5 Academic Primary Health Care, Uppsala, Sweden; 6 Department of Anatomy, Physiology and Biochemistry, Swedish University of Agricultural Sciences, Uppsala, Sweden

**Keywords:** Glutamate, Glutamate receptors, Mast cells, NMDA receptors, Tryptase, Neuroimmunology, Ion channel signalling

## Abstract

Mast cells are emerging as players in the communication between peripheral nerve endings and cells of the immune system. However, it is not clear the mechanism by which mast cells communicate with peripheral nerves. We previously found that mast cells located within healing tendons can express glutamate receptors, raising the possibility that mast cells may be sensitive to glutamate signaling. To evaluate this hypothesis, we stimulated primary mast cells with glutamate and showed that glutamate induced the profound upregulation of a panel of glutamate receptors of both the ionotropic type (NMDAR1, NMDAR2A, and NMDAR2B) and the metabotropic type (mGluR2 and mGluR7) at both the mRNA and protein levels. The binding of glutamate to glutamate receptors on the mast cell surface was confirmed. Further, glutamate had extensive effects on gene expression in the mast cells, including the upregulation of pro-inflammatory components such as IL-6 and CCL2. Glutamate also induced the upregulation of transcription factors, including Egr2, Egr3 and, in particular, FosB. The extensive induction of FosB was confirmed by immunofluorescence assessment. Glutamate receptor antagonists abrogated the responses of the mast cells to glutamate, supporting the supposition of a functional glutamate–glutamate receptor axis in mast cells. Finally, we provide in vivo evidence supporting a functional glutamate–glutamate receptor axis in the mast cells of injured tendons. Together, these findings establish glutamate as an effector of mast cell function, thereby introducing a novel principle for how cells in the immune system can communicate with nerve cells.

## Introduction

The pathophysiological mechanisms of pain and dysfunctional repair/regeneration, especially in soft connective tissues, are still unclear, and the debate continues regarding the role of inflammation in the disease process.^1^ In peripheral tissues, the peripheral nervous system plays key roles in regulating inflammation, pain responses, and the repair of damaged tissue via afferent and efferent pathways.^2^ Peripheral nerve endings at the site of injury can release potent neuropeptides (e.g., Substance P) with the ability to modify the function of fibroblast-like cells in injured tendon tissue.^3–5^ The released neuropeptides may affect the function of resident mast cells (MCs) and macrophages.^2,6^ Conversely, MCs contain substances that, when released from the granules, may alter the function of both the peripheral nervous system and tissue cells.^6,7^ It has been hypothesized that MCs residing near nerve endings may degranulate and thus affect the function of the peripheral nervous system, which makes them potential targets for modulating inflammation and pain. However, firm evidence for an interaction between MCs and afferent nerve endings, as well as the molecular mechanism(s) behind such interactions, remains to be established.

Glutamate is the major excitatory mediator of the nervous system^8,9^ and has been implicated in various pain conditions.^10^ NMDAR1, an ionotropic N-methyl-D-aspartate (NMDA) receptor, has been the focus of various pain studies, including those on tendinopathy.^11^ It is a heteromeric complex protein consisting of four subunits derived from three different protein families (NMDAR1, NMDAR2, and NMDAR3).^12^ However, the composition of these subunits can vary depending on different cell types and affects their functional properties.^3^ In patients with tendon pain, a 10-fold upregulation of NMDAR1 expression has been observed in transformed tenocytes, in the endothelial and adventitial layers of neovessel walls and in nerve fibers presumed to be sprouting.^13^ Nerve ingrowth, in combination with the expression of different pain signaling molecules, may be important for pain regulation in tendinopathy, tendon healing, and inflammation. Intriguingly, we recently showed that NMDAR1 was profoundly upregulated in a rat model of tendon rupture, and we noted that tendon injury was accompanied by extensive MC degranulation, suggesting that the injury may be related to activation of the MC compartment.^14^ Moreover, we demonstrated that NMDAR1 colocalized with MCs in the injured, but not the healthy, tendons.^14^

The above findings raise the intriguing possibility that glutamate receptor expression may be induced in MCs activated by nerve signaling mechanisms, and it is thus reasonable to assume that MC-expressed glutamate receptors may have a functional role in the communication between the cells of the peripheral nervous system and those of the immune system. Here, we evaluated this hypothesis and showed that glutamate induces robust expression of a panel of glutamate receptors in MCs. Moreover, we show that the neo-expressed glutamate receptors on MCs have a functional impact by mediating gene expression in the MCs exposed to glutamate. Finally, we provide in vivo evidence supporting a functional glutamate–glutamate receptor axis expressed by the MCs in injured tendons. Altogether, these findings show, for the first time, that MCs are able to respond to glutamate stimulation by a functional glutamate–glutamate receptor axis, thereby introducing a novel principle to explain how immune system cells communicate with nerve cells.

## Materials and methods

### Reagents

MK-801 was purchased from Abcam (Cambridge, UK), and (±)-α-Methyl-(4-carboxyphenyl)glycine (MCPG) was purchased from Sigma-Aldrich Sweden AB (Stockholm, Sweden).

### Generation and culture of bone marrow-derived MCs

Femoral and tibial bones from the hind legs of C57BL/6 male mice were used for isolation of bone marrow cells. The extracted cells were cultured in Dulbecco’s modified Eagle medium supplemented with 10% heat-inactivated fetal bovine serum, 60 µg/ml penicillin, 50 µg/ml streptomycin sulfate, 2 mM L-glutamine, and 30% WEHI 3B-conditioned media (which contains IL-3) as described.^15^ The cells were maintained at a concentration of 0.5–1 × 10^6^ cells/ml with weekly changes of the medium.

### In vitro stimulation and viability test

Mature MCs (4–8 weeks) were stimulated with L-glutamate (0, 10, 100, or 500 µM) for three different time periods (1, 4, and 72 h). Cell viability was determined as described.^16^

### Immunocytochemistry and histology

To monitor morphological effects on the MCs, cytospin slides were prepared using approximately 20–50 × 10^3^ cells/slide. The cells were stained with May-Grünwald (concentrated) and 2.5% Giemsa stains. MC degranulation (∼2 × 10^6^ cells in Tyrode’s buffer) was quantified by measuring the extent of the β-hexosaminidase release.^15^

### Immunofluorescence and confocal microscopy

To evaluate the expression and localization of glutamate receptors (NMDA-Rs) and MC tryptase, immunofluorescence was used. Cells were first placed onto glass slides and fixed with cold acetone (−20 °C) for 15 min. Staining with antibodies was performed following the protocol described.^14^ Briefly, the slides were first blocked with normal horse and/or goat serum, followed by a PBS wash (3×) and incubation overnight (at 4 °C, in the dark) with the following primary antibodies: rabbit monoclonal anti-NMDAR1 (Millipore C.N. AB 9864) and/or rabbit polyclonal against NMDAR1, rabbit polyclonal against NMDAR2A, NMDAR2B, mGluR1, mGluR2, mGluR7 (1:100, Abcam, Stockholm, Sweden), or glutamate (1:1000, MILLIPORE C.N. AB5018). For tryptase or glutamate staining, the cells were blocked with either normal horse serum (for single tryptase or glutamate staining) or normal goat serum (for double tryptase/NMDAR1 and/or glutamate/NMDAR1 staining), followed by PBS washes (3×). After the first blocking step, the tissue sections/primary cells were blocked to quench nonspecific binding of avidin (see the final step) using an avidin/biotin blocking kit (Vector Laboratories, Burlingame, CA). After washing, the sections/cells were incubated with another primary antibody overnight (4 °C in the dark). Single-staining for tryptase was performed using a mouse monoclonal anti-tryptase antibody (1:2000, MAB 1222, CHEMICON® International, Inc, Temecula, CA). For the double-staining experiments, a rabbit anti-tryptase antiserum was used (1:500 dilution). For FosB staining, rabbit monoclonal primary antiserum was used (1:800, Thermo Fisher Scientific, C.N. MA5-15056). All primary antibodies were diluted with PBS/1% BSA/0.3% Triton X-100 to permeabilize the cells. Next, the sections and/or cells were washed in PBS (3 × 5 min) and then incubated for 60 min (on a shaker) with a biotinylated secondary antibody. As a secondary antibody, biotinylated horse anti-mouse Ig (1:250 dilution in 1% PBS/1% BSA) was used for NMDAR1 staining and for the monoclonal anti-tryptase antibody. For detection of the rabbit anti-tryptase/NMDAR antibody, biotinylated goat anti-rabbit Ig (1:250 dilution in PBS/1% BSA) or horse anti-rabbit Ig was used as the secondary antibody. Finally, the sections/cells were incubated with streptavidin-Cy2 (for tryptase/glutamate; 1:2000, Amersham Int., Poole, UK) or streptavidin-Cy3 (for NMDAR1, NMDAR2A, NMDAR2B, mGluR1, mGluR2, and mGluR7/FosB; 1:5000, Amersham Int.). For double-staining, tryptase and NMDAR1 staining was performed consecutively to prevent nonspecific signals. For visualization of nuclei, DAPI (4,6-diamidino-2-phenylindole, Invitrogen) staining was performed. Digital images were captured using a Nikon fluorescence microscope (Nikon Eclipse 90i, Shinagawa-ku, Tokyo, Japan) equipped with a CCD camera (DS-Qi1 monochromatic digital camera). Glutamate/FosB and tryptase/FosB colocalization in the tissue and cells was monitored by using confocal and/or super resolution microscopy (Zeiss LSM700 or LSM710, Oberkochen, Germany) using a ZEN blue instrumental system (version 2.1; BioVis facilities, Uppsala University). All photographs were taken at original magnifications of ×200, ×400, or ×630 with an oil objective. All 3D images were captured with z-stacks and adjusted with IMARIS software (Bitplane, Zürich, Switzerland) for making 3D movies.

### ^3^H-glutamate binding assay

Assays for the binding of ^3^H-labeled glutamate (PerkinElmer, Stockholm, Sweden) to the MCs (1 × 10^6^ cells) were performed in 1.5-ml microcentrifuge tubes using 100 nM ^3^H-labeled glutamate (specific activity = 48.6 Ci/mmol) for different incubation periods (5, 20, 40, 60, and 180 min) at 4 °C. Incubation in the absence or presence of excess unlabeled glutamate (50 µM) was followed by the addition of ^3^H-labeled glutamate. These reactions were terminated with ice-cold Tyrode’s buffer (pH = 7.5) followed by filtration for 5 min (400 rcf). Specific binding was calculated by taking the cpm (counts per minute) corresponding to total binding (only ^3^H-labeled glutamate) and subtracting the cpm corresponding to the binding of ^3^H-glutamate in the presence of excess unlabeled glutamate. In addition, the MCs (1 × 10^6^ cells) were incubated with increasing doses of ^3^H-labeled glutamate (5, 25, 50, 100, and 400 nM) for 40 min followed by quantification of binding, as described above. The radioactivity was determined by scintillation counting after adding scintillation cocktail (PerkinElmer, Hägersten, Sweden) (1:2 volume).

### RNA isolation and qPCR

Cell pellets from the MCs (cultured at 10^6^ cells/ml) were used to purify total RNA. The cells were lysed with RNA lysis buffer (RA1) containing ß-mercaptoethanol following the NucleoSpin® RNA isolation protocol (MACHEREY-NAGEL GmbH & Co. KG). RNA purity and concentration were measured a NanoDrop™ 2000 spectrophotometer (Thermo Scientific™). Approximately 200 ng of RNA was used in RT-PCR for cDNA synthesis with an iScript^TM^ cDNA synthesis kit (Bio-Rad, Hercules, CA). The efficiency of each primer pair was checked by the iTaqTM Universal SYBR® Green (Bio-Rad), Supermix protocol. When a satisfactory primer efficiency (~80–110%) and dissociation curve (slope −3.1 to −3.6) was obtained, a qPCR reaction was run in duplicate with 384-well microtiter plates (Sarstedt, Nümbrecht, Germany) with 5 min of centrifugation (2000 × *g*) prior to qPCR (CFX384 Touch™, Bio-Rad). The cycling conditions were as follows: step 1: 95 °C (10 min); step 2: 95 °C (15 s); step 3: 60 °C (60 s); step 4: 72 °C (20 s). Steps 2–4 were repeated 40× with a dissociation stage (Bio-Rad).

### ELISA

IL-6, TNF-α, CCL2, CCL3 (Thermo Fisher Scientific), and CCL7 (eBioscience, Bender MedSystems GmbH, Vienna, Austria) were quantified by ELISA following the instructions of the respective manufacturers. High protein-binding ELISA plates (Sarstedt, Nümbrecht, Germany) were used, and the plates were read by an EMAX® plate reader (Molecular Devices, San Jose, CA).

### Microarray analysis

Transcriptome profiling was conducted using GeneChip™ Clariom D mouse array analysis (SciLifeLab, Uppsala University, Sweden). RNA quality was evaluated by using the Agilent 2100 Bioanalyzer system (Agilent Technologies Inc, Palo Alto, CA). We used 250 ng of total RNA to generate amplified and biotinylated sense-strand cDNA from the entire expressed genome according to the GeneChip™ WT PLUS reagent kit user manual (P/N 703174, Thermo Fisher Scientific Inc., Life Technologies, Carlsbad, CA). GeneChip™ ST Arrays (GeneChip™ Clariom D mouse array) were hybridized for 16 h in a 45 °C incubator and rotated at 60 rpm. According to the GeneChip® expression wash, stain, and scan manual (P/N 702731, Thermo Fisher Scientific Inc., Life Technologies), the arrays were then washed and stained using a GeneChip™ Fluidics Station 450 and finally scanned using the GeneChip™ Scanner 3000 7G (array and analysis facility protocol, SciLifeLab, Uppsala, Sweden).

### Animal model for tendon injury and tendinopathy

As previously described,^14^ an animal model for the Achilles tendon (AT) injury was used to evaluate the localization of the target biomarkers in the injured AT and compared the results with those of the control AT. The study was approved by the Linköping Animal Ethics Committee (ethical number 15-15) for animal experiments, and the institutional guidelines and protocols were followed for the care and treatment of laboratory animals.^17^

### Statistical analysis

All the data were analyzed with one- or two-way analyses of variance followed by Tukey’s multiple comparisons tests of the groups. The results were analyzed using GraphPad Prism 8.1.0 (GraphPad Software, CA). Student’s unpaired *t* test with Welch’s correction was used to analyze the experiments with two groups. *p*-Values were considered significant as *p < 0.05, **p < 0.01, ***p < 0.001, and ****p < 0.0001.

## Results

### Glutamate induces MC degranulation

**Fig. 1:**
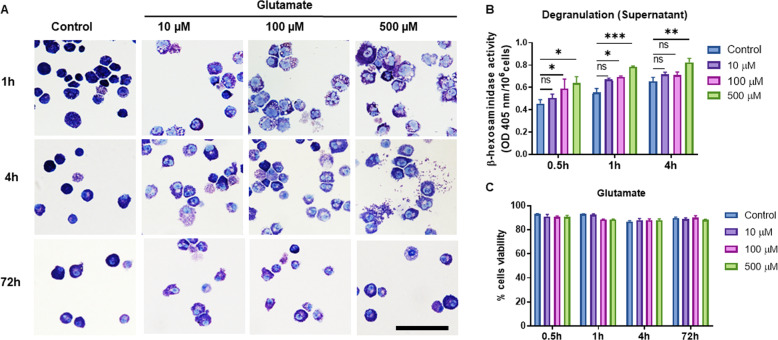
Glutamate induces MC degranulation. **a** MCs were treated with glutamate at the concentrations and time periods indicated, followed by preparation of cytospin slides and staining with toluidine blue. **b** MCs were treated with glutamate as indicated, and the amount of released β-hexosaminidase was measured. **c** Cell viability of the glutamate-treated MCs. Data in **b** and **c** represent the means ± SEM (*n* = 3). *p ≤ 0.05; **p ≤ 0.01; ***p ≤ 0.001. Scale bars: 25 μm

To investigate whether glutamate functionally affects MCs, we first assessed whether glutamate influences MC morphology. To this end, we cultured primary MCs, exposed the cells to various concentrations of glutamate, and evaluated their morphological characteristics and levels of released β-hexosaminidase (a granule marker enzyme). As shown in Fig. 1a, the untreated MCs showed a typical rounded morphological appearance and intense granular staining. However, after exposure to glutamate, distinct morphological changes were noted, as indicated by the appearance of expelled granules and a general fading in granular staining intensity, suggesting partial degranulation. In line with this observation, we noted a significant increase in the levels of extracellular β-hexosaminidase in the MC cultures exposed to glutamate (Fig. 1b). To rule out that these effects were associated with cell toxicity, we assessed cell viability. As seen in Fig. 1c, glutamate concentrations up to 500 µM incubated with the MCs for as long as 72 h, induced no significant toxicity of the MCs. For the remaining experiments, nontoxic doses of glutamate were used.

### Glutamate induces the expression of glutamate receptors in the MCs

**Fig. 2:**
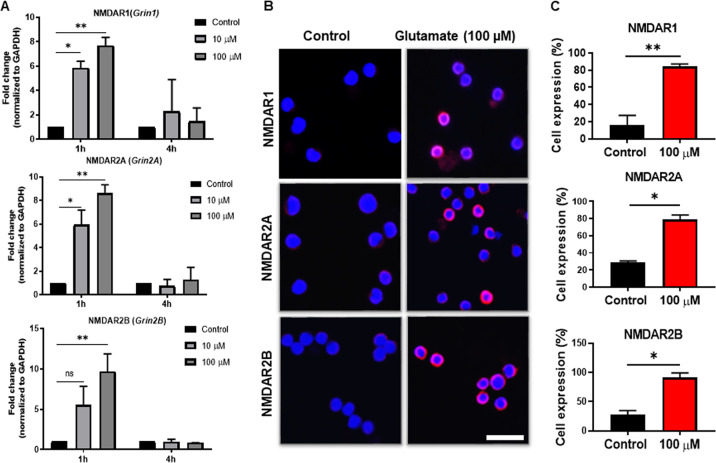
Glutamate induces ionotropic NMDA receptors at the mRNA and protein levels. **a** MCs (1 × 10^6^/ml) were exposed to glutamate (10 or 100 µM) for either 1 h or 4 h, followed by the measurement of the mRNA encoding NMDAR1, NMDAR2A, and NMDAR2B by qPCR. **b** Immunofluorescence staining for NMDAR1, NMDAR2A, and NMDAR2B in the MCs incubated for 24 h with glutamate (100 µM). **c** Quantification of the immunofluorescence staining, presented as the means ± SEM (*n* = 3). Significance was calculated with an unpaired *t*-test with Welch’s correction. *p ≤ 0.05; **p ≤ 0.01. Scale bars: 25 μm

The biological effects of glutamate are mediated by binding to glutamate receptors, which can be of either the ionotropic type (linked to ion channels) or metabotropic type (G protein-coupled) type.^18,19^ Hence, the observed effects of glutamate on the MCs imply either that the MCs express glutamate receptors at baseline or, alternatively, that glutamate receptor expression can be induced by exposure of the MCs to glutamate. To investigate these possibilities, we first focused on the expression of various ionotropic glutamate receptors. As shown in Fig. 2a, low expression of the ionotropic glutamate receptor NMDAR1 (Grin1) was observed in the MCs under baseline conditions (control group). However, after stimulation with glutamate, a strong induction of the NMDAR1 (Grin1; ~8-fold, *p* ≤ 0.01) gene was observed. Notably, NMDAR1 induction was transient and significant after 1 h of glutamate stimulation, whereas no NMDAR1 induction was observed after 4 h of stimulation. This pattern of induction was also observed for two other ionotropic glutamate receptors, NMDAR2A and NMDAR2B (Fig. 2a). To verify the glutamate-induced upregulation of these ionotropic glutamate receptors at the protein level, we stained for the corresponding receptors using fluorescence microscopy (Fig. 2b). Indeed, glutamate induced high and significant expression of the NMDAR1, NMDAR2A, and NMDAR2B proteins (Fig. 2c). Isotype control antibodies produced negligible staining, showing that the staining of these receptors was specific (not shown).

**Fig. 3:**
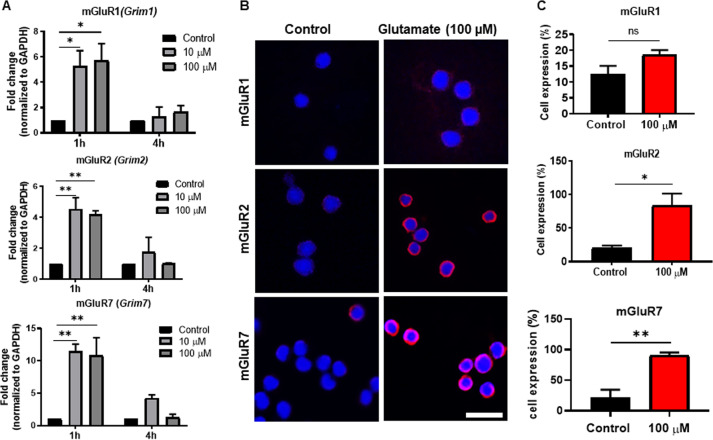
Glutamate induces metabotropic NMDA receptors at the mRNA and protein levels. **a** MCs (1 × 10^6^/ml) were exposed to glutamate (10 or 100 µM) for either 1 h or 4 h, followed by measurement of the mRNA encoding mGluR1 (Grim1), mGluR2 (Grim2), and mGluR7 (Grim7) by qPCR. **b** Immunofluorescence staining for mGluR1, mGluR2, and mGluR7 in the MCs incubated for 24 h with glutamate (100 µM). **c** Quantification of immunofluorescence staining, presented as the means ± SEM (*n* = 3). The significance was calculated with an unpaired *t*-test with Welch’s correction. *p ≤ 0.05; **p ≤ 0.01. Scale bars: 25 μm

Next, we assessed whether glutamate could also induce the expression of metabotropic glutamate receptors. As seen in Fig. 3a, glutamate stimulation of the MCs caused a robust upregulation of the mGluR1 (Grim1) gene; mGluR2 (Grim2) and mGluR7 (Grim7) were also induced to a similar extent at the mRNA level. Similar to the induction of the ionotropic receptors, the induction of all of these metabotropic glutamate receptors was transient, being high at 1 h, whereas only an upregulation trend was seen after 4 h of glutamate stimulation. At the protein level, clear induction was observed for mGluR2 and mGluR7 by using fluorescence microscopy, whereas only weak induction of mGluR1 was observed (Fig. 3b). Quantification of the staining revealed that the induction of mGluR2 and mGluR7 was statistically significant, whereas a trend of upregulation was seen for mGluR1 (Fig. 3c).

### Glutamate binds to glutamate receptors on the MC surface

**Fig. 4:**
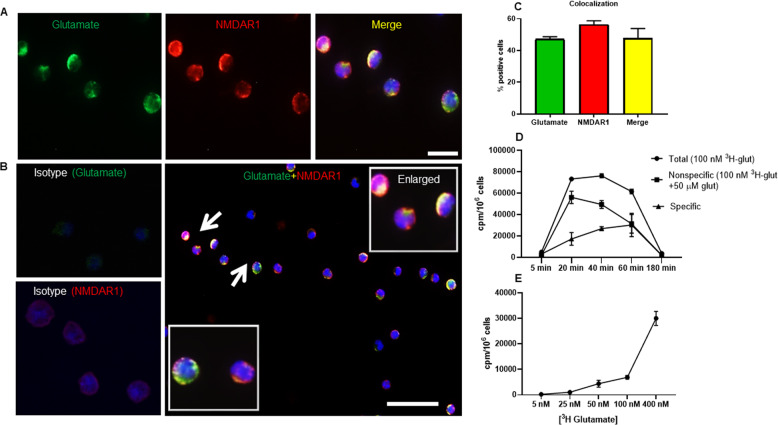
Colocalization of glutamate and NMDAR1 in the cell membrane of the glutamate-treated MCs. **a** Immunofluorescence staining of glutamate-treated MCs showing colocalization of glutamate and NMDAR. DAPI was used as a nuclear marker. MCs were double positive for glutamate and NMDAR1. **b** Isotype controls for glutamate and NMDAR1 staining are shown, revealing the specificity of the staining. The panel also shows an overview of the stained cells, with enlarged areas showing colocalization of glutamate and NMDAR1. **c** Fraction of the cells that are positive for glutamate, NMDAR1 and double positive for glutamate and NMDAR1, respectively. Arrows show the association between glutamate and NMDAR1. Scale bars: 25 μm (**a**) and 50 μm (**b**). **d** Binding of ^3^H-labeled glutamate to the MCs. MCs (1 × 10^6^ cells) were incubated with 100 nM ^3^H-glutamate for the time periods indicated. MCs were incubated in the absence or presence of excess unlabeled glutamate as indicated. Specific binding is presented as total binding (^3^H-labeled glutamate only) minus the binding of ^3^H-glutamate in the presence of excess unlabeled glutamate. **e** MCs (1 × 10^6^ cells) were incubated for 40 min with the indicated concentrations of ^3^H-glutamate in the presence of 50 µM of unlabeled glutamate. **d**, **e** Cells were harvested, washed, and subjected to scintillation counting. Results are shown as the mean of triplicate determinations (±SEM) and are representative of at least two individual experiments

The findings presented above reveal that glutamate induces the expression of a panel of glutamate receptors in MCs. Next, we evaluated if the glutamate was physically associated with its corresponding receptors on the MC surface. To this end, we costained glutamate-treated MCs for glutamate and NMDAR1. These analyses revealed that glutamate was indeed found on the surface of the MCs (Fig. 4a, b). Furthermore, strong colocalization (>50% positive cells) of glutamate and its corresponding receptor, i.e., NMDAR1, was observed (Fig. 4c). We also performed experiments in which the binding of radiolabeled glutamate to the MCs was assessed. As seen in Fig. 4d, e, radiolabeled glutamate bound to the MCs in a time- (Fig. 4d) and concentration-dependent (Fig. 4e) manner. These findings reveal that glutamate interacts with the MCs by binding to glutamate-induced glutamate receptors on the MC surface.

### Glutamate influences the gene expression profile in the MCs

**Fig. 5:**
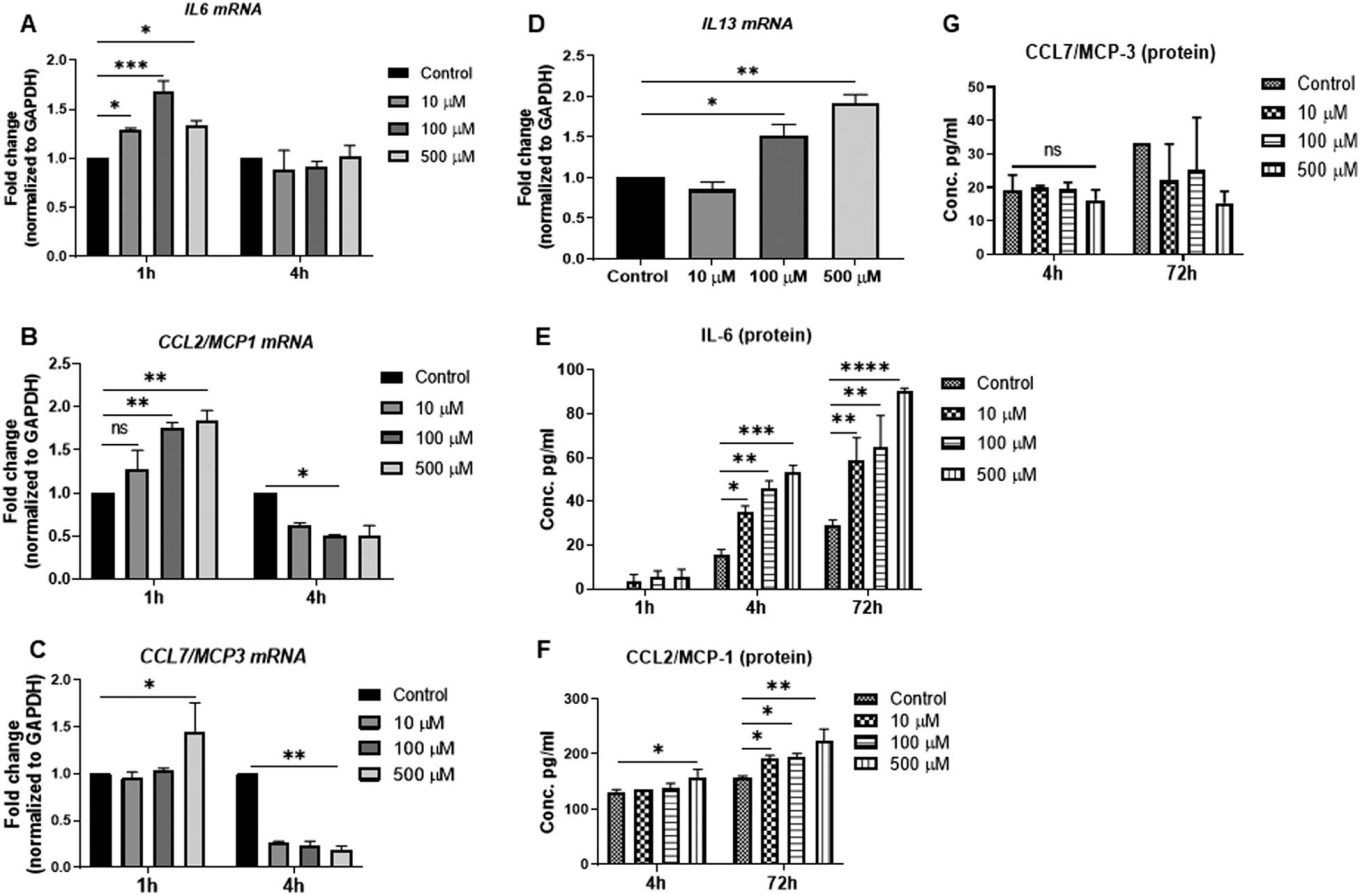
Glutamate induces pro-inflammatory responses. MCs (1 × 10^6^/ml) were exposed to glutamate (10, 100, or 500 µM) for 1 h, 4 h, or 72 h. **a**–**d** Relative mRNA levels (fold change) corresponding to pro-inflammatory markers IL-6, CCL2/MCP-1, CCL7/MCP-3, and IL-13 were quantified by qPCR. The mRNA levels were normalized to the levels of the housekeeping gene GAPDH. **e**–**g** Protein levels of IL-6, CCL2/MCP-1, and CCL7/MCP-3 were measured by ELISAs. Data are presented as the mean values ± SEM from at least two independent experiments. Significance was calculated with one-way and/or two-way ANOVA with Tukey’s multiple comparisons test. *p ≤ 0.05; **p ≤ 0.01

In the next set of experiments, we evaluated the functional consequences of glutamate–MC interactions beyond the observed effects on degranulation (see Fig. 1). For this purpose, we used a gene array screen to evaluate the possibility that glutamate affects gene expression patterns in the MCs. Based on this approach, we observed that the expression of a large number of genes was induced to varying extent in the glutamate-stimulated MCs (Supplementary Fig. 1). Notably, several of these glutamate-induced genes corresponded to pro-inflammatory products. To validate these findings, we used qPCR. As shown in Fig. 5a–d, glutamate caused a significant upregulation of the genes encoding IL-6, CCL2, CCL7, and IL-13, which are all cytokines/chemokines. At the protein level, the induction of IL-6 and CCL2 was validated with ELISAs (Fig. 5e, f), whereas no significant effect on CCL7 protein was observed (Fig. 5g). We also used ELISAs to evaluate whether glutamate stimulation of the MCs caused the release of TNF-α or CCL3, but no significant effects on these compounds was detected.

**Fig. 6:**
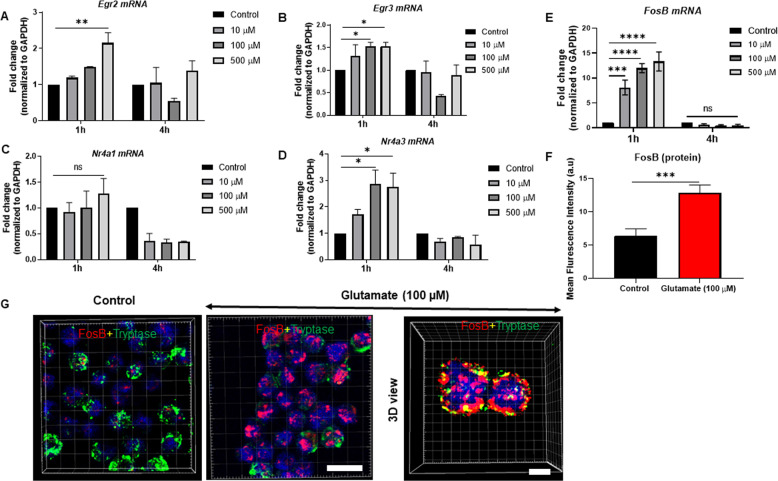
Glutamate induces the expression of transcription factors and nuclear receptors. MCs (1 × 10^6^/ml) were exposed to glutamate (10, 100, or 500 µM) for 1 h, 4 h, or 24 h. **a**–**e** Relative mRNA levels (fold change) for Egr2, Egr3, Nr4a1, Nr4a3, and FosB were quantified by qPCR. The relative mRNA levels of targets were normalized to the housekeeping gene GAPDH. **g** Visualization of FosB protein in control- and glutamate-treated MCs using confocal microscopy. Cells were costained for FosB, tryptase (MC granule marker), and DAPI (nuclear marker). **f** Quantification of FosB staining in control and glutamate-treated MCs. Scale bars: 25 μm. Data represent mean values ± SEM (*n* = 3). Significance was calculated with an unpaired *t*-test with Welch’s correction. ***p ≤ 0.001

The gene array screen also indicated that glutamate induced the expression of the nuclear receptors Nr4a1 and Nr4a3 and the transcription factors Egr2 and Egr3 (Supplementary Fig. 1). Indeed, the upregulated expression of Egr2, Egr3, and Nr4a3 in glutamate-stimulated MCs was verified by the qPCR analysis (Fig. 6a, b, d), whereas the upregulation of Nr4a1 was not significant (Fig. 6c). As judged by the gene array screen (see Supplementary Fig. 1), the transcription factor FosB was one of the genes showing the most pronounced upregulation after glutamate stimulation, and we therefore focused further on this gene. As shown in Fig. 6e, we indeed verified a pronounced upregulation of FosB in response to glutamate, even at low concentrations of glutamate (10 µM). A robust induction of FosB protein was also validated through confocal microscopy of FosB stained with an antibody (Fig. 6g). Quantification of the staining intensity confirmed a significant induction of FosB protein in the glutamate-stimulated MCs (Fig. 6f). Notably, in the unstimulated MCs, FosB staining showed mostly cytosolic, diffuse localization. In contrast, FosB staining was predominantly nuclear in the glutamate-stimulated cells (Fig. 6f). In these analyses, tryptase was used as an MC marker, and glutamate stimulation caused a reduction in tryptase staining (Fig. 6g; see also Supplementary Video 1). The latter finding is most likely explained by the release of tryptase as a consequence of the MC degranulation in response to glutamate (see also Fig. 1).

### The effects of glutamate on the MCs are reversed by glutamate receptor antagonists

**Fig. 7:**
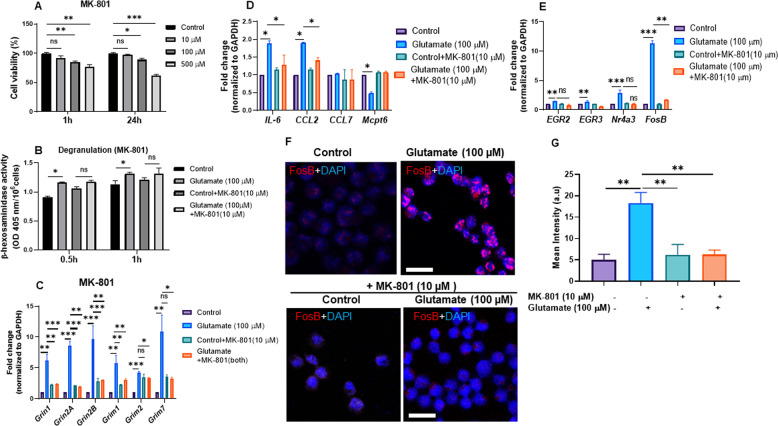
Effects of glutamate receptor antagonists on MC responses to glutamate. **a** MCs (1 × 10^6^/ml) were incubated with MK-801 (10, 100, 500, or 1000 µM) for 1 or 24 h, followed by assessment of cell viability. **b**–**e** MCs (1 × 10^6^/ml) were untreated or treated for 1 h with either glutamate, MK-801, or glutamate + MK-801. Effects on β-hexosaminidase release (degranulation) (**b**), expression of glutamate receptors (**c**), pro-inflammatory genes (**d**), nuclear receptors and transcription factors (**e**) were measured. Data represent mean values ± SEM (*n* = 3). Significance was calculated with an unpaired *t*-test with Welch’s correction. **f** Confocal images showing the effect of MK-801 on FosB protein intensity in the control- and glutamate-treated MCs. **g** Mean intensity of the FosB protein was quantified from representative images using ZEN 2.1 software. Scale bars: 25 μm. *p ≤ 0.05; **p ≤ 0.01; ***p ≤ 0.001

To provide further evidence for an impact of glutamate on MC function, we asked whether the effects of glutamate on MC parameters could be reversed by glutamate receptor antagonists. To this end, we first used MK-801, a broad range NMDAR receptor antagonist. To evaluate its potential toxicity, we incubated MCs with various concentrations of MK-801 and then assessed cell viability. This experiment revealed that MK-801 was nontoxic to the MCs at 10 µM for as long as, at least, 24 h, whereas limited but significant toxicity was observed at higher doses (Fig. 7a). To address the impact of glutamate receptor inhibition on MC function, we therefore used MK-801 at 10 µM. As shown in Fig. 7b, there was no significant glutamate-induced increase in β-hexosaminidase release in the presence of MK-801, suggesting that the inhibition of the NMDA receptors had a dampening effect on MC degranulation. Next, we evaluated whether MK-801 affected the mRNA expression of various glutamate receptors. These experiments confirmed a strong upregulation of glutamate receptor expression in response to glutamate (Fig. 7c). In contrast, no significant or negligible effects on glutamate receptor expression was observed in the presence of MK-801 (Fig. 7c). Further, MK-801 blunted the induction of the various pro-inflammatory genes that were induced by glutamate stimulation (Fig. 7d). In addition, MK-801 caused a reduction in the expression of tryptase (Mcpt6), a granule marker in MCs (Fig. 7d). Further analysis showed that the glutamate receptor antagonist significantly blunted the induction of Egr2, Egr3, Nr4a3, and FosB caused by glutamate (Fig. 7e). At the protein level, MK-801 abrogated the induction of the FosB that had been observed in the glutamate-stimulated MCs (Fig. 7f, g).

We then analyzed whether inhibition of metabotropic glutamate receptors had an impact on the downstream responses of the MCs stimulated with glutamate. These experiments showed that MCPG, an antagonist of metabotropic glutamate receptors, was nontoxic to the MCs at concentrations as high as 500 µM after incubation for as long as 1 h, whereas significant toxicity was seen after 24 h at concentrations >5 µM (Supplementary Fig. 2a). Further analysis revealed that MCPG (at nontoxic conditions), similar to the antagonist of the ionotropic glutamate receptors, had dampening effects on the β-hexosaminidase release from the glutamate-stimulated MCs (Supplementary Fig. 2b). Further, MCPG, similar to MK-801, caused a reduction in the expression of glutamate receptor mRNA in response to glutamate stimulation of the MCs (Supplementary Fig. 2c).

### FosB colocalizes with glutamate and is expressed by the MCs after tendon injury in vivo

**Fig. 8:**
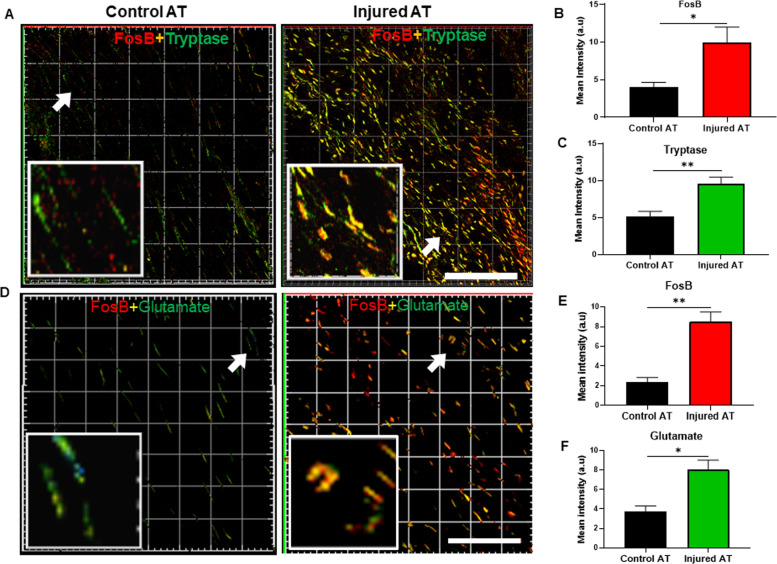
Colocalization of FosB, MC tryptase, and glutamate in an injured tendon. **a** Immunofluorescence image showing colocalization of FosB and MC tryptase (Mcpt6) in an injured Achilles tendon (AT). Note the abundance of cells double positive for tryptase (MCs) and FosB in the injured, but not in control, tissues. **b**, **c** Quantification of FosB and tryptase staining in the control and injured ATs. **d** Immunofluorescence images showing colocalization of FosB and glutamate in the control and injured ATs. Note the abundance of cells double positive for FosB and glutamate in the injured, but not in the control, ATs. **e**, **f** Quantification of FosB and glutamate staining in the control and injured tendons. *p ≤ 0.05; **p ≤ 0.01. Scale bars: 100 μm

In our final set of experiments, we evaluated the in vivo significance of our findings by focusing on the possibility that MCs in injured tissue might show upregulated expression of FosB and sought to determine whether such FosB induction was linked to a glutamate–glutamate receptor axis. To this end, we used injured and control tendon tissues from rats subjected to AT rupture, and we costained these samples for the MC marker tryptase and for FosB. As shown in Fig. 8a, c (see also Supplementary Video 2), we noted a profound increase in the MCs localized to the injured (healing) tendon, as indicated by tryptase staining. Moreover, we noted higher FosB staining intensity in the injured tendons than we observed in the control tendons (Fig. 8a, b). Tryptase and FosB staining also clearly showed strong colocalization in injured tendon (Fig. 8a; see also Supplementary Video 3and 4), i.e., indicating that FosB is expressed, to a large extent, within the MC compartment. To assess whether the induction of FosB in the MCs was related to glutamate stimulation, we additionally stained for glutamate. Indeed, as shown in Fig. 8d–f, high levels of glutamate and FosB colocalization was observed in the injured tendons. Altogether, these findings suggest that glutamate is associated with the stimulation of the MCs during tendon healing in vivo with a marked upregulation in FosB, findings that are in clear agreement with the data presented above.

## Discussion

MCs are versatile immune cells that contribute to a large panel of pathological conditions. Most notably, MCs are strongly implicated in allergic conditions, but there is also evidence that MCs contribute to various autoimmune diseases, fibrosis, cancer, and metabolic disorders.^20–25^ In addition, there is emerging evidence suggesting that MCs may potentially be involved in conditions associated with neurogenic inflammation, as exemplified by a proposed role for MCs in mediating inflammation, pain, and tissue healing.^6,26–28^

In support of functional MC-nerve communication, MCs are frequently found in close association with nerve endings.^26–28^ However, the mechanisms by which MCs may interact with peripheral nerves have been mostly elusive. One scenario is that secretory products released from MCs can interact with nerve endings. Such products include histamine and serotonin, which may interact with corresponding receptors on afferent neurons.^6^ Moreover, it has been proposed that tryptase, a serine protease found in large quantities in MC granules,^7^ can cleave and thereby activate protease-activated receptor 2 (PAR2) expressed by afferent neurons, thereby triggering nerve signaling.^29–31^ It has also been suggested that MCs and nerves can communicate in the opposite direction, i.e., from the nerves to MCs. In this scenario, nerve endings, activated by MC products, release neuropeptides (e.g., substance P and calcitonin gene-related peptide), which activate MCs, thereby providing an loop that amplifies the cascade of MC activation/nerve signaling.^6^ However, although these scenarios are conceivable, there is still little firm evidence that such pathways are operative in vivo.

Here, we explored a fundamentally novel principle for how MCs may respond to nerve cells. In a previous report, we found that glutamate receptors were upregulated in vivo in AT rupture and subsequent healing. We found that glutamate receptors were expressed to a large extent by the MCs located within the injured tendon, whereas the MCs in uninjured tendons showed low or nondetectable glutamate receptor expression.^14^ Prompted by these findings, we hypothesized that glutamate may have an impact on MCs, and here, we evaluated this possibility. Intriguingly, we show that glutamate induced the expression of a panel of glutamate receptors on the MC surface. It is known that the concentration of glutamate in blood is ~50–100 µM, whereas the extracellular levels of glutamate in the tissues are considerably lower (<1 µM).^32–34^ Our data suggest that MC activation was triggered at glutamate concentrations ≥10 µM. It is thus reasonable to assume that the baseline concentration of extracellular glutamate in tissues is below this level. However, upon tissue injury or inflammatory conditions, it is likely that the glutamate concentration increases to levels sufficient for MC triggering. This MC induction may take place either due to glutamate release from peripheral nerve endings or through extravasation from the blood due to increased vascular permeability.

Interestingly, the induction of glutamate receptor expression was completely blocked by a broad range NMDAR-class glutamate receptor antagonist and by an antagonist of metabotropic glutamate receptors. Altogether, these findings imply that glutamate initially binds to glutamate receptors that are expressed at a low level on the MC surface, in turn causing signaling that strongly amplifies the cell surface expression of the various glutamate receptors. Another interesting observation indicated that glutamate receptor induction was transient, being high at 1 h but substantially weakening 4 h after glutamate stimulation, which implies a quick and transient function of the glutamate–glutamate receptor axis in the MCs. This finding is in general agreement with the role of MCs in other types of settings, e.g., allergic activation and in the defense against envenomation, where MCs predominantly contribute at early stages through mechanisms associated with rapid degranulation and the release of granule mediators.^35^

In addition to inducing the expression of glutamate receptors, exposure of the MCs to glutamate activated the expression of a number of other genes, including genes associated with several inflammatory compounds. It is therefore possible that signaling through a glutamate–glutamate receptor axis in the MCs contributes to neurogenic inflammation and the inflammatory reaction that accompanies tendon healing and other similar conditions. It is plausible that the cytokines/chemokines released through this mechanism may contribute, either directly or indirectly, to the modulation of tissue healing and pain signaling in such settings. The induction of these genes was completely abrogated in the presence of both a broad range and a metabotropic glutamate receptor antagonist, providing further evidence that the induction of these genes was a direct consequence of signaling mediated by the glutamate–glutamate receptor axis.

Among other genes found to be induced by glutamate was FosB, which was upregulated to a higher level than most other glutamate-induced genes in the MCs. FosB is a transcription factor that dimerizes with Jun family members, thereby forming AP-1 complexes that are implicated in a wide variety of settings, such as cell proliferation, differentiation, and apoptosis.^36^ In particular, FosB has been implicated in drug addiction,^37^ but there are also reports suggesting that FosB can have an impact on neurogenesis,^38^ osteoblastoma,^39^ regulation of cyclooxygenase-2 expression,^40^ transforming growth factor β1 signaling,^41^ and regulation of complement receptor expression.^42^ Elevated FosB expression has been previously observed in an AT rupture model.^43^ In the latter study, Egr2 was upregulated in the injured tendon, in line with our observed upregulation of Egr2 in the glutamate-stimulated MCs. Hence, we propose a scenario in which glutamate receptor expression is induced after initial exposure of the MCs to glutamate released from neurons, followed by the induction of FosB expression as a consequence of the signaling events downstream of the glutamate–glutamate receptor interaction. The downstream consequences of FosB upregulation most likely include activated transcription of target genes. FosB expression could thereby contribute to the induction of gene expression observed in the glutamate-stimulated MCs. In agreement with this hypothesis, a previous report suggested that Nrf1 in complex with FosB activates TNF expression in a murine MC-like cell line.^44^

Altogether, this study has identified a novel mechanism by which cells in the peripheral nervous system can interact with those in the immune system. A potential effect of this activation of MCs through the glutamate–glutamate receptor axis may be the modulation of neurogenic inflammation, tissue healing and, possibly, pain signaling. This supposition is in line with previous studies suggesting a role for MCs in mediating pain responses.^28^ However, further studies are required to fully establish the role of glutamate signaling in MCs in mediating physiological and pathophysiological responses in healing tissues and to evaluate whether the glutamate–glutamate receptor axis in MCs can be exploited for therapeutic purposes.

## Supplementary information

Suppl. Video 1

Suppl. Video 2

Suppl. Video 3

Suppl. Video 4

Suppl. Fig. 1

Suppl. Fig. 2
