# Supplementary material for: Glutamate triggers the expression of functional ionotropic and metabotropic glutamate receptors in mast cells
Source: Cell Mol Immunol. 2020 Apr 20;18(10):2383–92. doi: 10.1038/s41423-020-0421-z (PMC8484602; doi:10.1038/s41423-020-0421-z)
Supplement: Supplementary file 5 — Suppl. Fig. 1 [file 41423_2020_421_MOESM5_ESM.pdf]

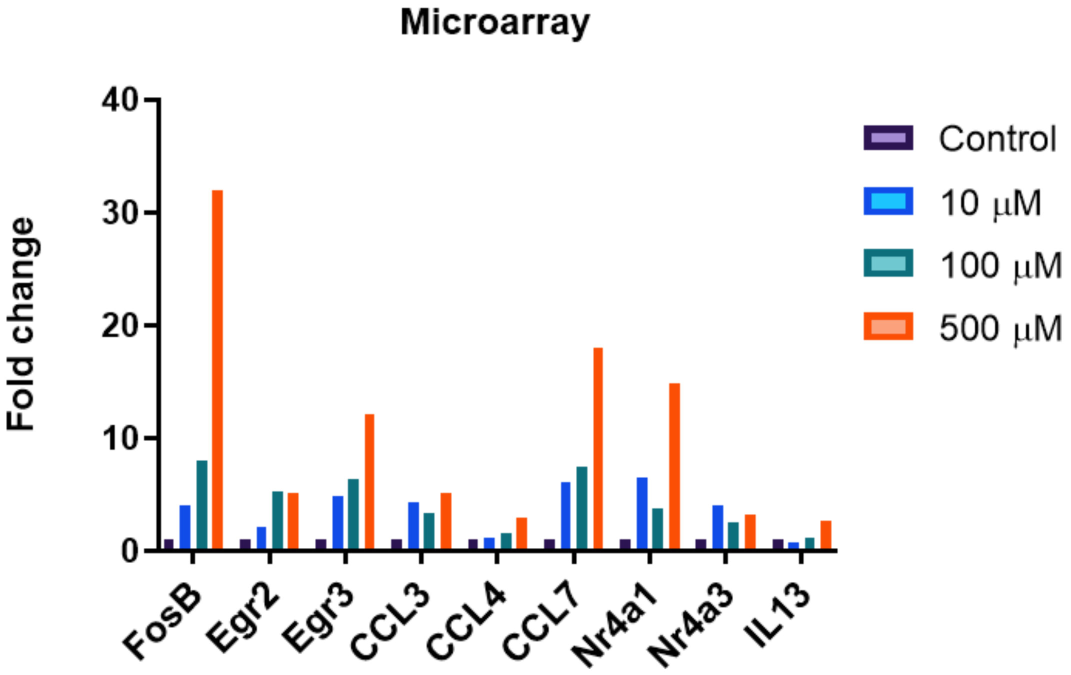

Suppl. Fig. 1. Upregulation of genes in mast cells stimulated with glutamate. Primary mast cells were stimulated with glutamate at the indicated concentrations. Total RNA was recovered and used for Affymetrix gene array analysis. The figure depicts extent of upregulation of selected genes.
