# Supplementary material for: Glutamate triggers the expression of functional ionotropic and metabotropic glutamate receptors in mast cells
Source: Cell Mol Immunol. 2020 Apr 20;18(10):2383–92. doi: 10.1038/s41423-020-0421-z (PMC8484602; doi:10.1038/s41423-020-0421-z)
Supplement: Supplementary file 6 — Suppl. Fig. 2 [file 41423_2020_421_MOESM6_ESM.pdf]

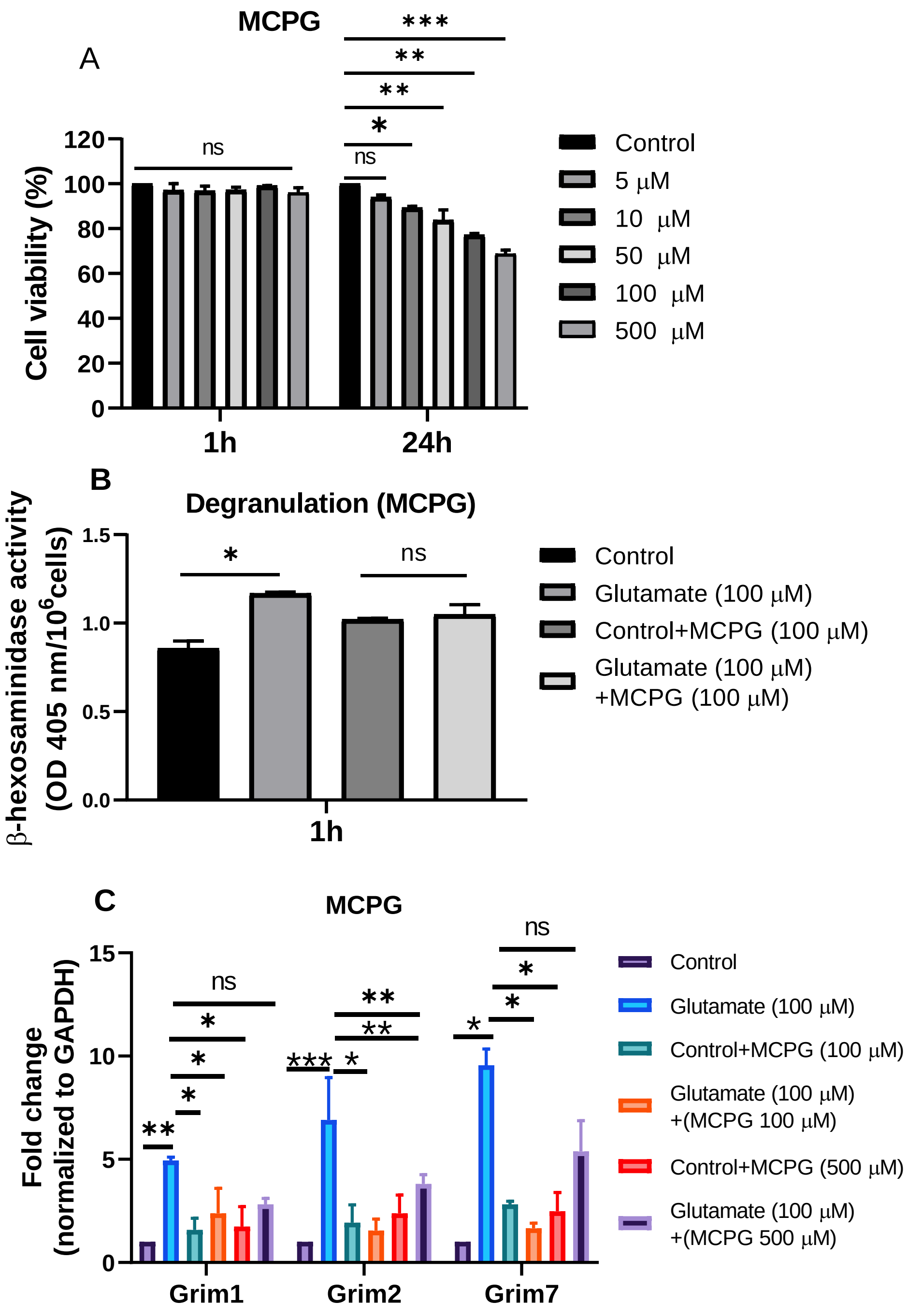

Supplemental Figure 2. Effect of glutamate receptor antagonism on MC responses to glutamate. (A) MCs ($1x10^6$/ml) were incubated with MCPG at the indicated concentrations for 1h or 24h, followed by assessment of cell viability. (B-C) MCs (1 x 10^6/ml) were untreated or treated for 1h with either glutamate, MCPG (antagonist of metabotropic glutamate receptors) or glutamate + MCPG. (B) Effects on β-hexosaminidase release (degranulation) (B) and (C) expression of mRNA coding for glutamate receptors were measured. Data are presented as mean values ± SEM from at least two independent experiments. The statistical significances were calculated with one-way and/or two-way ANOVA with Tukey's multiple comparisons test. *p ≤ 0.05, **p ≤ 0.01.
